# Human umbilical cord mesenchymal stem cell-derived extracellular vesicles attenuate experimental autoimmune encephalomyelitis via regulating pro and anti-inflammatory cytokines

**DOI:** 10.1038/s41598-021-91291-3

**Published:** 2021-06-02

**Authors:** Samira Ahmadvand Koohsari, Abdorrahim Absalan, Davood Azadi

**Affiliations:** 1 Department of Biology, Marvdasht Branch, Islamic Azad University, Marvdasht, Iran; 2 Department of Medical Laboratory Sciences, Khomein University of Medical Sciences, Ghods Avenue, Khomein, Markazi Province 38818-58573 Iran

**Keywords:** Immunology, Molecular biology, Neuroscience, Stem cells

## Abstract

The therapeutic effects of mesenchymal stem cells-extracellular vesicles have been proved in many inflammatory animal models. In the current study, we aimed to investigate the effect of extracellular vesicles (EVs) derived from human umbilical cord-MSC (hUCSC-EV) on the clinical score and inflammatory/anti-inflammatory cytokines on the EAE mouse model. After induction of EAE in C57Bl/6 mice, they were treated intravenously with hUCSC-EV or vehicle. The clinical score and body weight of all mice was registered every day. On day 30, mice were sacrificed and splenocytes were isolated for cytokine assay by ELISA. Cytokine expression of pro-/anti-inflammatory cytokine by real-time PCR, leukocyte infiltration by hematoxylin and eosin (H&E) staining, and the percent of glial fibrillary acidic protein (GFAP) and myelin basic protein (MBP) positive cells by immunohistochemistry were assessed in the spinal cord. Our results showed that hUCSC-EV-treated mice have lower maximum mean clinical score (MMCS), pro-inflammatory cytokines, and inflammatory score in comparison to the control mice. We also showed that hUCSC-EV administration significantly improved body weight and increased the anti-inflammatory cytokines and the frequency of Treg cells in the spleen. There was no significant difference in the percent of GFAP and MBP positive cells in the spinal cord of experimental groups. Finally, we suggest that intravenous administration of hUCSC-EV alleviate induce-EAE by reducing the pro-inflammatory cytokines, such as IL-17a, TNF-α, and IFN-γ, and increasing the anti-inflammatory cytokines, IL-4 and IL-10, and also decrease the leukocyte infiltration in a model of MS. It seems that EVs from hUC-MSCs have the same therapeutic effects similar to EVs from other sources of MSCs, such as adipose or bone marrow MSCs.

## Introduction

Multiple sclerosis (MS) is a chronic, autoimmune mediated and demyelinating disease of the central nervous system (CNS)^1^. It is a T-cell mediated autoimmune diseases, however, other immune cells such as B cells, macrophages, and microglia may play role in the pathogenesis of the disease^2^. Experimental autoimmune encephalomyelitis (EAE) is an animal model of inflammatory disease of the CNS, such as MS^3^. Several strategies have been proposed for the treatment of MS. Many of them are immunomodulatory agents that reduce the inflammatory responses in the CNS^4^. Many new approaches are under clinical trials and animal models of MS, such as stem cells. Among stem cells, mesenchymal stem cells (MSCs) have been considered in animal models and MS patients, and promising results have been observed^5^. MSCs are stromal cell progenitors and contain huge expansion potency. These cells can be isolated from different tissues, such as adipose tissue, bone marrow and umbilical cord blood^6^. In cell therapy, there are always concerns about malignancies or immune rejection^7^. In many studies, it has been shown that MSCs exert their effects on a paracrine manner, especially by their extracellular vesicles (EVs)^8^. EVs are membrane surrounded vesicles and generally referred to as exosomes, microvesicles, and apoptotic bodies with a size range of 30–1000 nm^7^. The effect of MSC-extracellular vesicle (MSC-EV) on inflammatory diseases have been demonstrated in many different animal models, such as liver disease^9^, experimental autoimmune uveitis^10^, kidney diseases^11^, and neurological disorders^12,13^. Since MSC-EVs have immunomodulatory effects and MS is an inflammatory disease in which the immune cells can cause demyelination by damaging oligodendrocytes, it seems that MSC-EVs are promising therapeutic agents for MS^14^. The effects of MSC-EVs on animal models of MS have been demonstrated in few studies. Farinazzo et al. have shown that intravenous (I.V.) administration of nanovesicles from adipose stem cells (NV-ASC) does not ameliorate established EAE, however, administration before the disease onset significantly decrease the spinal cord inflammation, demyelination and severity of EAE^15^. Garcia et al. have also investigated the effect of I.V. administration of MSC-EVs from human adipose tissue in a progressive model of MS, Theiler’s murine encephalomyelitis virus (TMEV)-induced demyelinating disease. Their results showed that I.V. administration of MSC-EVs reduced brain atrophy, decreased inflammatory infiltrates in the spinal cord, improved motor deficits, and increased cell proliferation in the subventricular zone in TMEV infected mice^16^. In another study, the effects of I.V. administration exosome from MSCs stimulated by interferon (IFN)-γ have been studied by Riazifar et al. and they showed MSC-Exo reduced demyelination and the mean clinical score of EAE mice, upregulated the number of T regulatory (Treg) cells in spinal cord, and decreased neuroinflammation in EAE mouse model^17^. In a recent study, Jafarinia et al. have studied the therapeutic effects of I.V. administration of MSC-EVs from human adipose tissue and showed that the maximum mean clinical score and myelin oligodendrocyte glycoprotein (MOG)-induced proliferation of splenocytes in MSC-EV-treated mice were significantly lower than the control mice; furthermore the inflammation score and the percentages of demyelination areas in MSC-EV-treated groups significantly decreased compared with the untreated control group^14^. Based on the above mentioned studies, in the current study, we assessed the effect of EVs derived from human umbilical cord-MSC (hUCSC-EVs) on clinical score and inflammatory/anti-inflammatory cytokines on EAE mouse model.

## Results

### hUCSC and hUCSC-EV characterization

**Figure 1:**
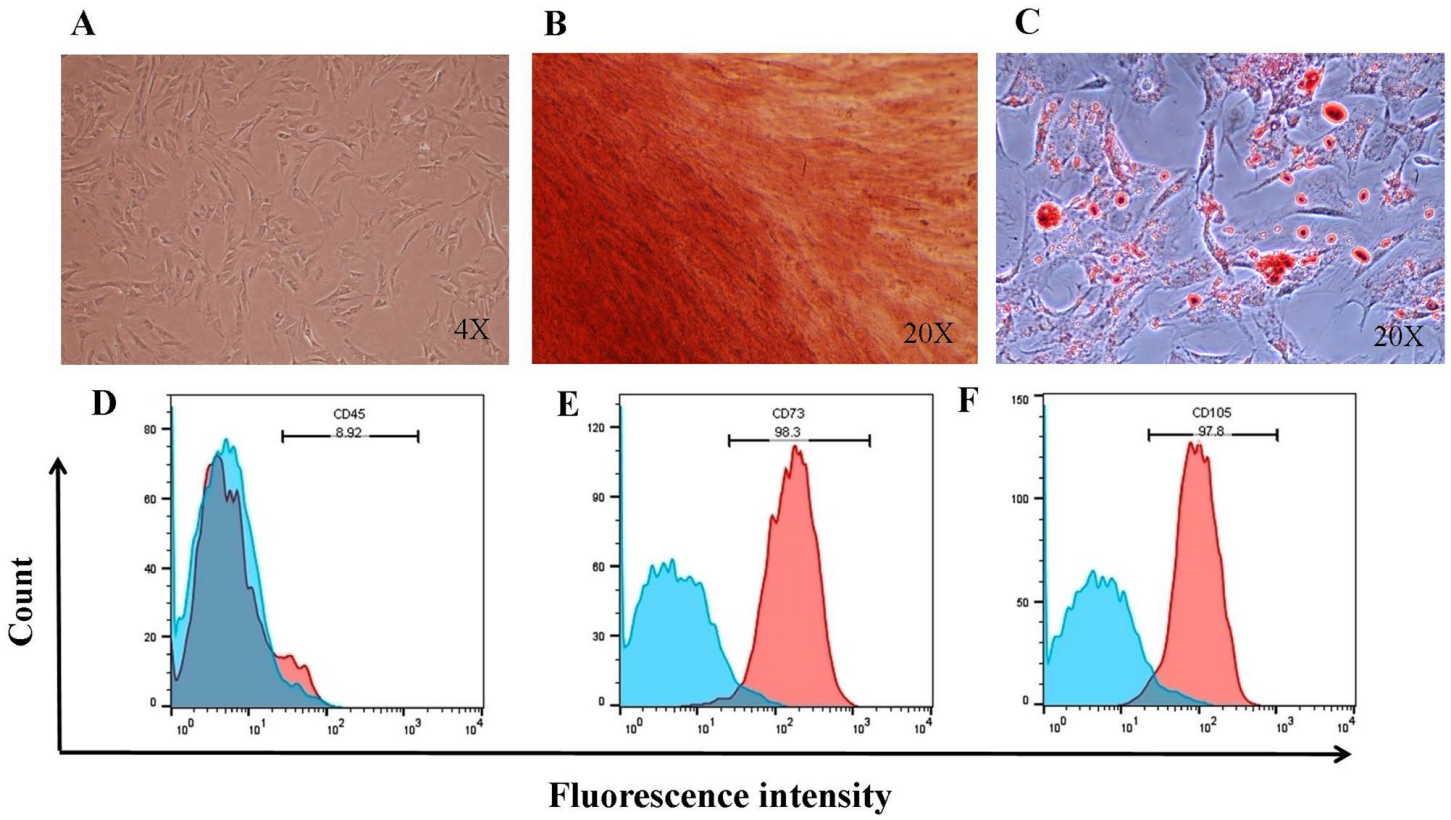
Characterization of hUCSCs. hUCSCs displayed adherent and fibroblast-like morphology (**A**). Alizarin red staining for osteogenic differentiation (**B**) and Oil red O staining for adipogenic differentiation (**C**). hUCSCs were analyzed for specific surface markers by flow cytometry, which confirmed lack of CD45 (**D**) and the expression of CD73 (**E**), CD105 (**F**). *hUCSC* human umbilical cord mesenchymal stem cell.

Our results confirmed that hUCSCs displayed adherent and fibroblast-like morphology (Fig. 1A). We also evaluated the hUCSCs for differentiation in to osteocytes (Fig. 1B) and adipocytes (Fig. 1C) using Alizarin red and Oil red O, respectively. Alizarin red staining demonstrated large amounts of calcium deposits and considerable number of lipid droplets after 20 days post adipogenic differentiationin hUCSCs by Oil red O staining. We also examined hUCSCs for expression of surface markers and confirmed lack of CD45 expression (Fig. 1D) and considerable expression of CD73 (Fig. 1E) and CD105 (Fig. 1F).

**Figure 2:**
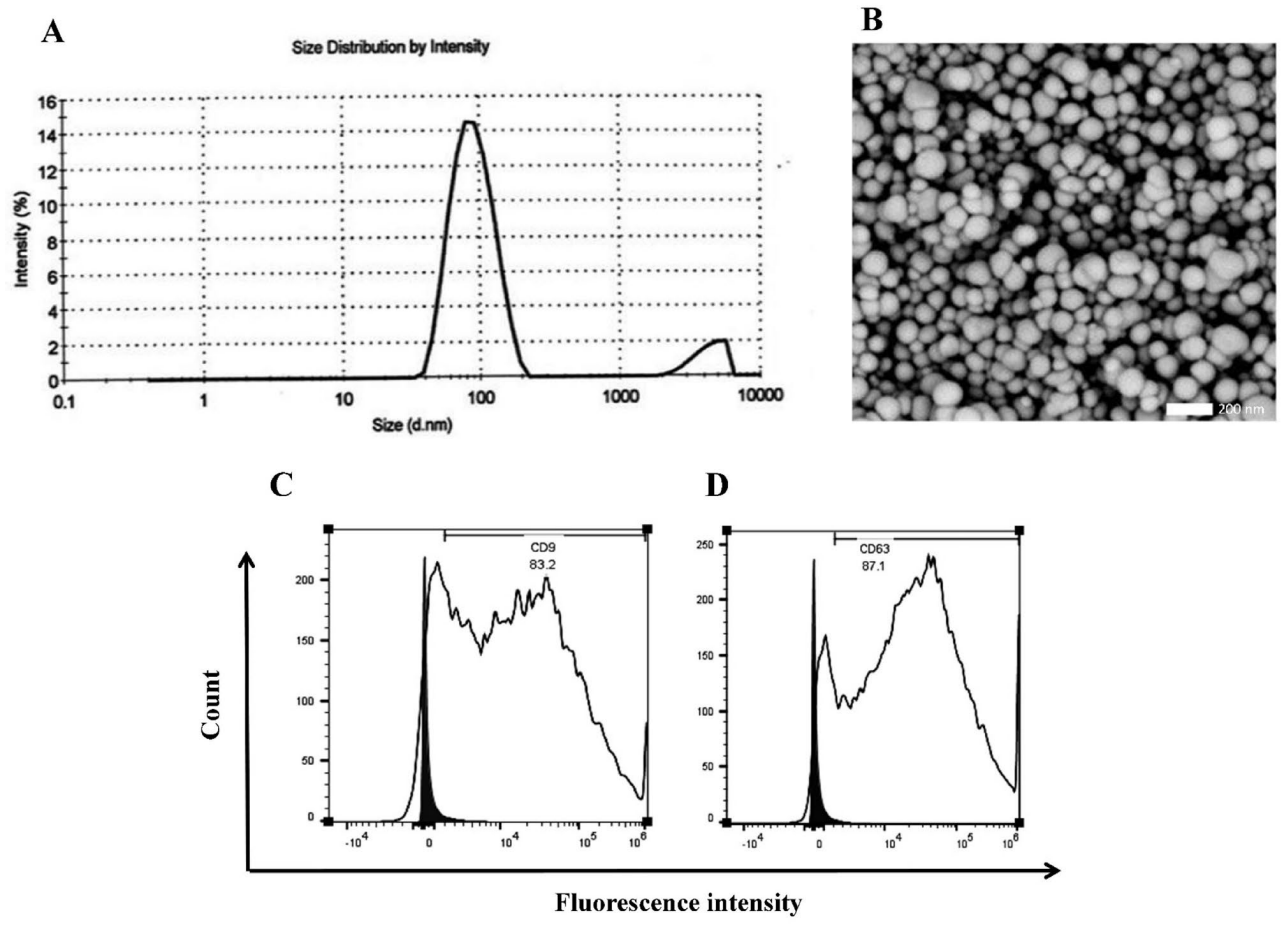
Characterization of hUCSC-EVs. The results of DLS showed that 91.2% of the solution ingredients had an average diameter of 90.89 nm (**A**). Electron microscopy confirmed EV’s size and shape (**B**). Flow cytometry analysis confirmed that hUCSC-EVs expressed CD9 (**C**) and CD63 (**D**). *DLS* dynamic light scattering, *hUCSC-EV* human umbilical cord mesenchymal stem cell-extracellular vesicle.

For the characterization of hUCSC-EVs, we examined the size of EVs using dynamic light scattering (DLS) and it showed that 91.2% of the solution ingredients had an average diameter of 90.89 nm (Fig. 2A). hUCSC-EV’s size and shape confirmed by electron microscopy (Fig. 2B). By flow cytometry analysis, we confirmed that hUCSC-EVs were positive for EV’s markers such as CD9 (Fig. 2C) and CD63 (Fig. 2D).

### hUCSC-EVs treatment reduce mean clinical score and increase the body weight in EAE mice

**Figure 3:**
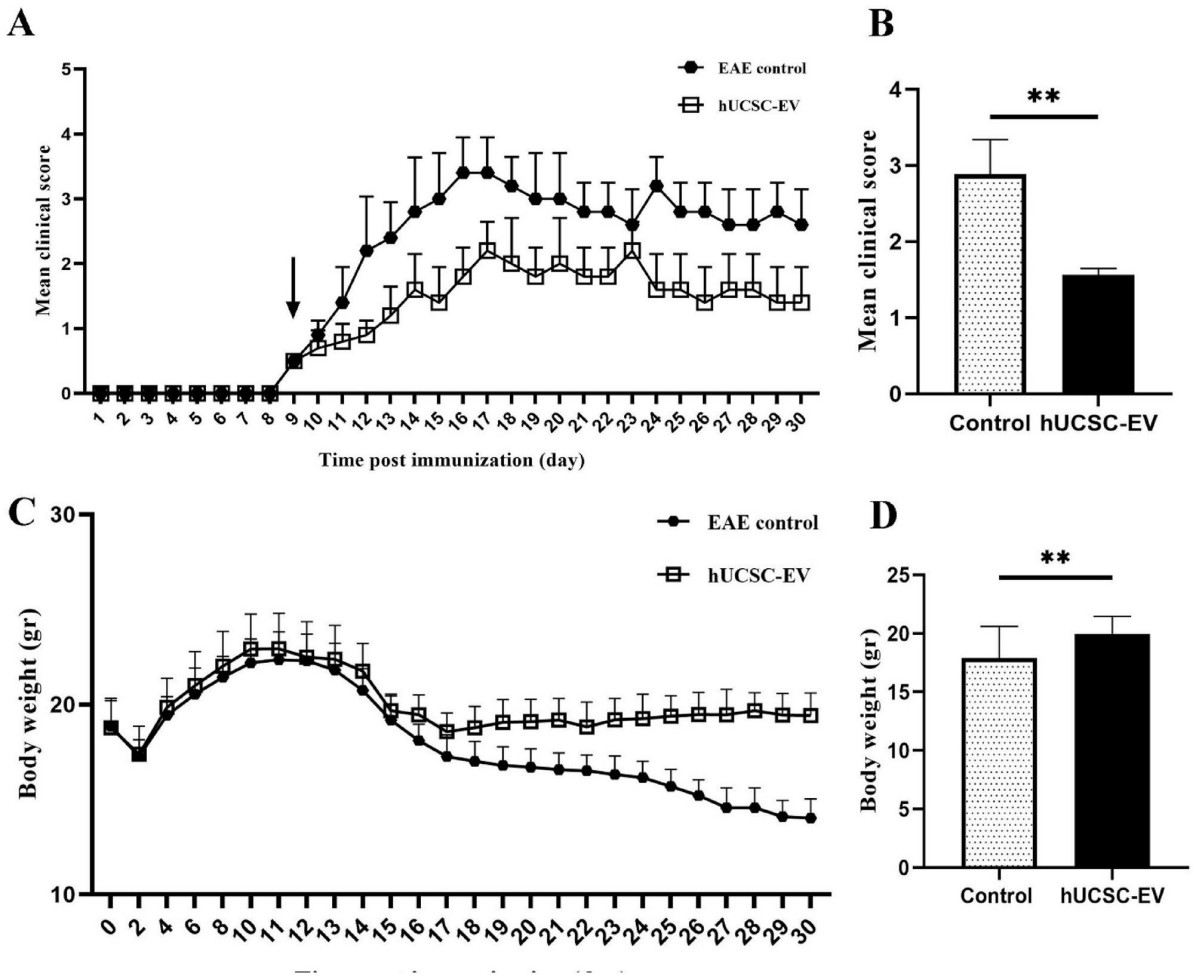
Comparison of the clinical score and body weight between hUCSC-EVs-treated and EAE control mice. MOG_35–55_-immunized C57Bl/6 mice were treated with PBS (EAE control) or hUCSC-EVs on day-9 post immunization (black arrow) (**A**). The MMCS of hUCSC- and MSC-EVs-treated mice were significantly lower than the MMCS of EAE control mice (**B**). The body weight of hUCSC-EVs-treated mice were significantly higher than the body weight of EAE control mice (**C**,**D**). Data are shown as mean ± SD. **p < 0.01. *EAE* experimental autoimmune encephalomyelitis, *hUCSC-EV* human umbilical cord mesenchymal stem cell-extracellular vesicle, *MMCS* maximum mean clinical score, *SD* standard deviation.

The clinical score of mice was monitored every day. The first clinical sign of EAE observed after 9 days of immunization. Our results showed that the maximum mean clinical score (MMCS) of hUCSC-EVs-treated mice was significantly lower than the MMCS of EAE control mice (p < 0.01) (Fig. 3A,B). We also evaluated the body weight of mice every day and the results showed that the body weight of hUCSC-EVs-treated mice was significantly higher than the body weight of EAE control mice (p < 0.01) (Fig. 3C,D). Overall, the MMSC and body weight of hUCSC-EV-treated mice were improved compared to the untreated EAE group (Table 1).

**Table 1:** Clinical and pathological features of EAE-treated mice. The parameters of hUCSC- and hUCSC-EVs-treated mice compared to control mice.

Groups	No.	Clinical score	Body weight	H&E	MBP	GFAP	% Treg cells
Control	10	2.89 ± 0.45	17.91 ± 2.7	2.8 ± 0.83	5.48 ± 1.31	4.03 ± 0.32	3.3 ± 1.05
hUCSC-EV	10	1.56 ± 09**	19.98 ± 1.5**	1.4 ± 0.54*	6.52 ± 0.65	3.78 ± 1.12	5.37 ± 0.87

*GFAP* glial fibrillary acidic protein, *hUCSC-EV* human umbilical cord-derived mesenchymal stem cell-extracellular vesicle, *H&E* hematoxylin and eosin, *MBP* Mmyelin basic protein, *SD* standard deviation, *Treg* T regulatory.

**p < 0.01.

*p < 0.05.

### hUCSC-EVs treatment modulate the pro- and anti-inflammatory cytokines balance

**Figure 4:**
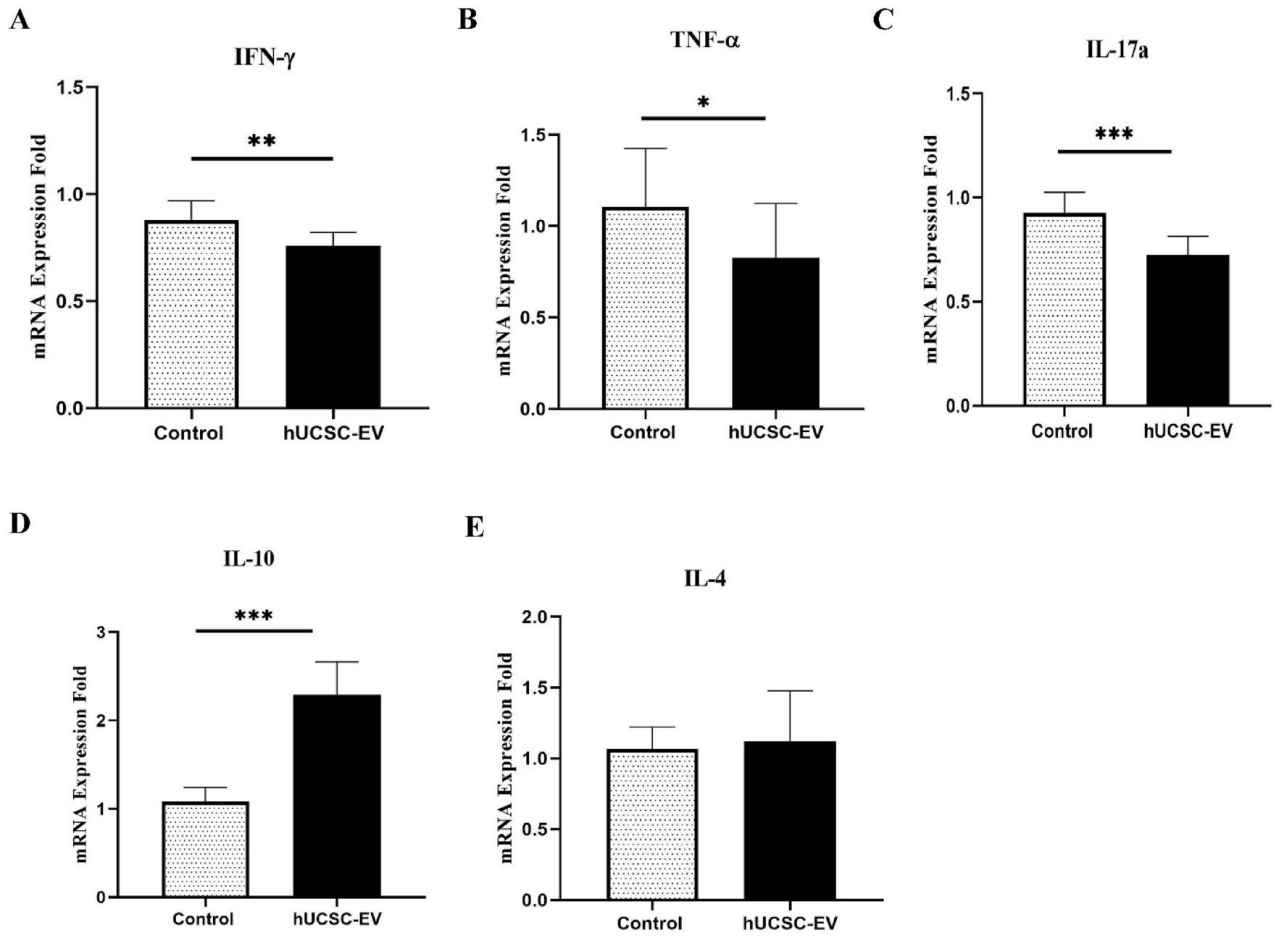
Differential expression of the pro-/anti-inflammatory cytokine genes in the CNS of experimental groups. Quantitative RT-PCR data demonstrated the downregulation of pro-inflammatory cytokines [IFN-γ (**A**), TNF-α (**B**), and IL-17a (**C**)] and upregulation of IL-10 (**D**) as anti-inflammatory cytokine in hUCSC-EVs-treated mice compared to EAE control mice. Data are shown as mean ± SD. ***p < 0.001, **p < 0.01, and *p < 0.05. *EAE* experimental autoimmune encephalomyelitis, *hUCSC-EV* human umbilical cord mesenchymal stem cell-extracellular vesicle, *IFN* interferon, *IL* interleukin, *SD* standard deviation, *TNF* tumor necrosis factor.

Immunomodulatory effects of hUCSC-EVs on the EAE mice were evaluated by measuring the pro/anti-inflammatory cytokines expression in the experimental groups using real-time RT-PCR. Our results showed that IFN-γ, TNF-α, and IL-17a significantly downregulated in the hUCSC-EVs-treated group compared to EAE control mice (p < 0.01, p < 0.05, and p < 0.001, respectively). On the other hand, IL-10 significantly upregulated in EVs-treated group in comparison to EAE control mice (p < 0.001). There was no significant difference in the expression of IL-4 between the groups (Fig. 4).

### The effect of hUCSC-EVs treatment on the secretion of cytokines by splenocytes

**Figure 5:**
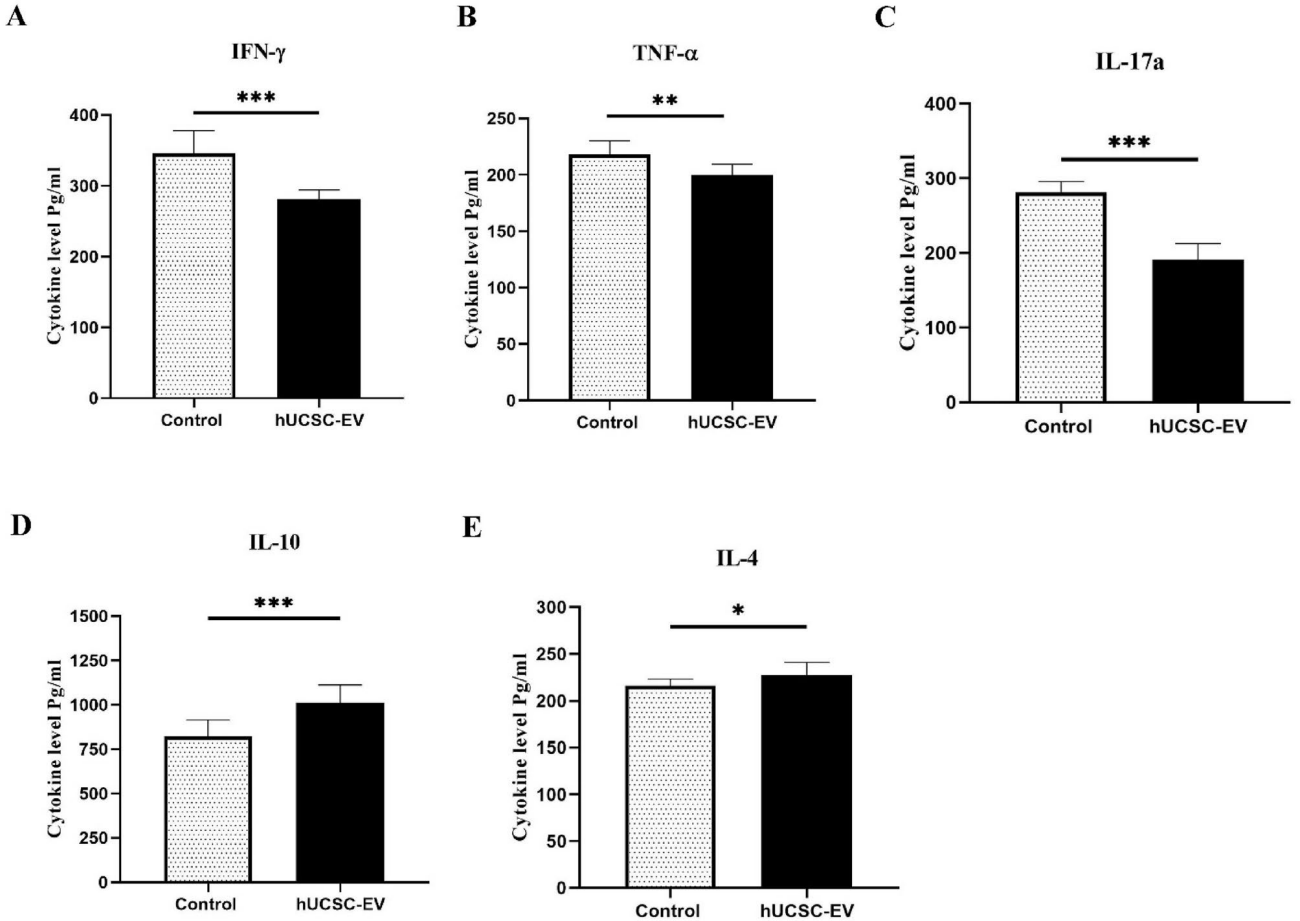
Analysis the secretion of pro-/anti-inflammatory cytokines by splenocytes in experimental groups. The mean levels of pro-inflammatory [IFN-γ (**A**), TNF-α (**B**), and IL-17a (**C**)] and anti-inflammatory cytokines [IL-10 (**D**) and IL-4 (**E**)]. Data are shown as mean ± SD. ***p < 0.001, **p < 0.01, and *p < 0.05. *hUCSC-EV* human umbilical cord mesenchymal stem cell-extracellular vesicle, *IFN* interferon, *IL* interleukin, *SD* standard deviation, *TNF* tumor necrosis factor.

To explain the beneficial effects of hUCSC-EVs on the clinical and pathological signs of EAE, the pro-inflammatory (TNF-α, IFN-γ, and IL-17a) and anti-inflammatory (IL-10 and IL-4) cytokines were also assessed in splenocyte cultures after being restimulated with MOG. Similar to results of pro-inflammatory cytokines expression, the production of IFN-γ, TNF-α, and IL-17a significantly decreased by splenocytes from hUCSC-EVs-treated mice compared to EAE control mice (p < 0.001, p < 0.01, and p < 0.001, respectively). Our results also demonstrated that the amount of anti-inflammatory cytokines (IL-10 and IL-4) production was significantly increased by splenocytes from hUCSC-EVs-treated mice compared to EAE control mice (p < 0.001 and p < 0.05, respectively) (Fig. 5).

### hUCSC-EVs treatment reduce leukocyte infiltration in spinal cord of EAE mice

**Figure 6:**
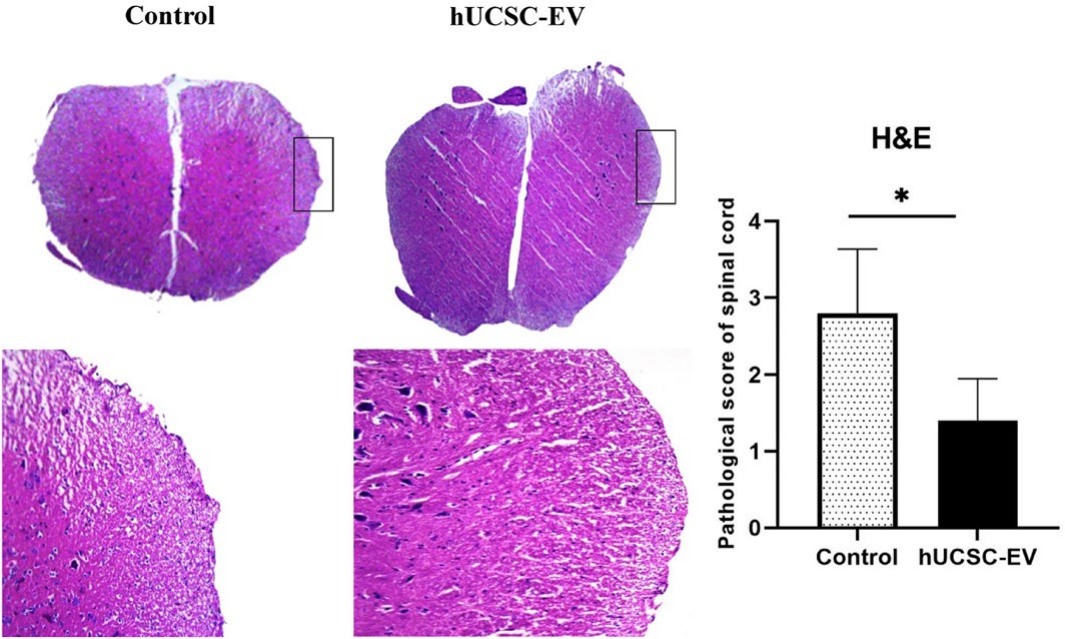
H&E staining in the spinal cord. Leukocyte infiltration into the spinal cord of untreated control, hUCSC-EVs-treated groups were assessed using H&E stating. Data are shown as mean ± SD. *p < 0.05. *H&E* hematoxylin and eosin, *hUCSC-EV* human umbilical cord-derived mesenchymal stem cell-extracellular vesicle, *SD* standard deviation.

Based on the immunomodulatory effect of hUCSC-EVs, we evaluated the leukocyte infiltration in spinal cord of experimental groups by hematoxylin and eosin (H&E) staining. Consistent with the clinical score results, the inflammation score in hUCSC-EVs-treated group significantly declined in comparison to EAE control mice (p < 0.05) (Fig. 6, Table 1).

**Figure 7:**
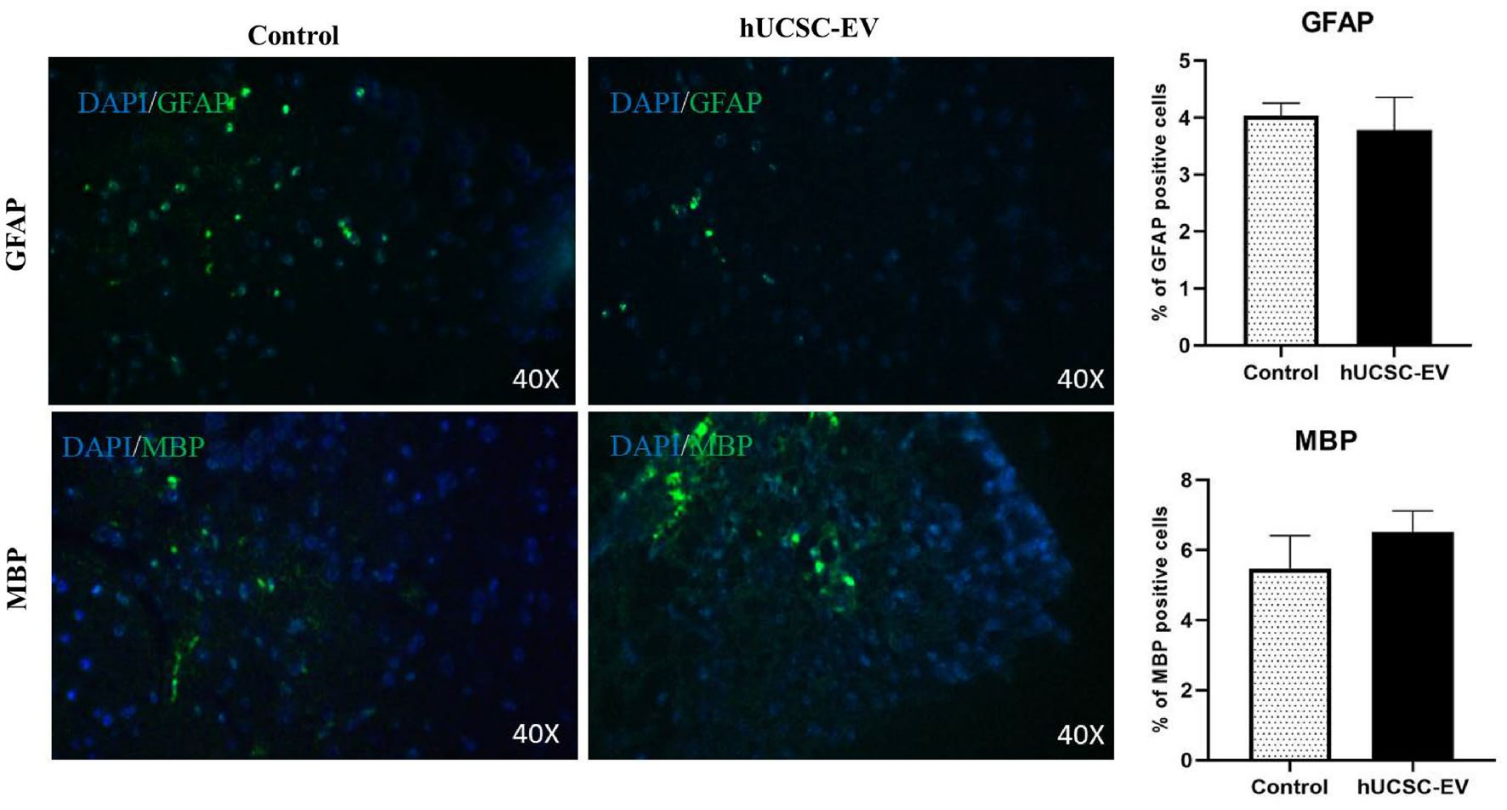
Immunofluorescence of repair-associated markers. There was no significant difference in the percent of positive MBP and GFAP cells between the untreated control and hUCSC-EV-treated groups by immunofluorescence. Data are shown as mean ± SD. *GFAP* glial fibrillary acidic protein, *MBP* myelin basic protein, *MFI* mean fluorescence intensity, *hUCSC-extracellular vesicle* human umbilical cord-derived mesenchymal stem cell-extracellular vesicle.

We also examined two repair-associated markers, GFAP and MBP, in experimental groups. Unfortunately, no significant difference was observed in the percent of these two markers in the spinal cord experimental groups (Fig. 7, Table 1).

### hUCSC-EVs treatment increase the frequency of Treg cell in spleen of EAE mice

**Figure 8:**
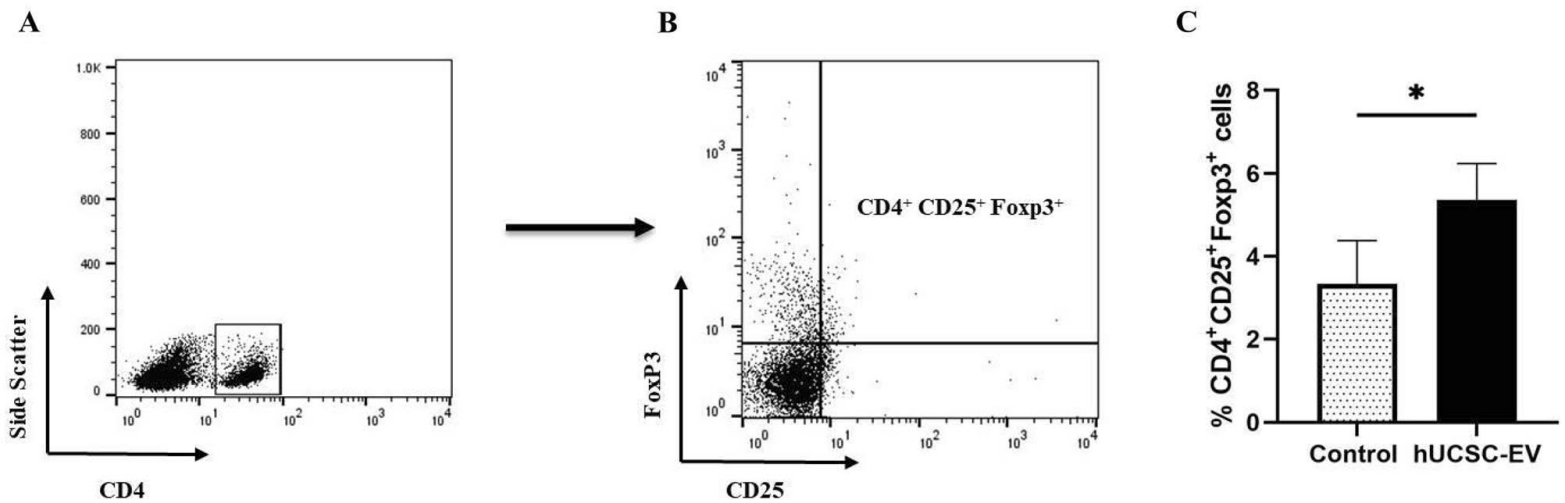
Frequency of CD4^+^CD25^+^Foxp3^+^ Treg cells in the spleen. CD4^+^ cells gated in lymphocyte gate (**A**) and CD25^+^Foxp3^+^ cells were determined in CD4^+^ gate (**B**). The frequency of CD4^+^CD25^+^Foxp3^+^ Treg cells in the spleen of hUCSC-EVs-treated mice was significantly higher than EAE control mice (**C**). Data are shown as mean ± SD. *p < 0.05. *hUCSC-EV* human umbilical cord-derived mesenchymal stem cell-extracellular vesicle, *Treg* regulatory T cells.

To evaluate the frequency of Terg cells in the spleen of EAE mice, we gated the lymphocytes and then, CD4^+^ cells were determined in lymphocyte gate. In CD4^+^ gate, we were assessed CD25^+^Foxp3^+^ cells. Our results showed that the frequency of CD4^+^CD25^+^FoxP3^+^ Treg cells in spleen of hUCSC-EVs-treated mice was significantly higher than EAE control mice (p = 0.03) (Fig. 8, Table 1).

## Discussion

The immunomodulatory function of MSCs has been shown in many human and animal model studies and they have proposed as potential therapeutic candidate for inflammatory and autoimmune diseases^18^. There are always concerns and limitations about stem cell therapy, such as cellular rejection, malignancies (tumor formation), difficulty in quality control of MSCs, and high cost^7^. However, in recent studies, it has been proposed that MSCs exert their effect on a paracrine manner by their EVs. Many studies have also demonstrated that MSC-EVs exert same therapeutic effects similar to their parent cells (MSCs)^7,14^. The effects of MSCs-EVs have been proven in many animal models, such as liver diseases^19,20^, EAU^21^, cardiovascular diseases^22^, kidney injury^11,23,24^, GVHD^25^, and some neurological diseases such as brain injury^26,27^ and EAE^16,17,28,29^.

In the current study, we aimed to investigate the therapeutic effects of hUCSC-EVs on EAE mice that are an animal model of MS disease. Our results showed that administration of EVs from mesenchymal stem cells isolated from human umbilical cord significantly improved the score and body weight of EAE mice. Our result is in line with other studies which have shown the therapeutic effects of MSC-EVs with different sources in animal models of MS. Riazifar et al. have shown that I.V. administration of Exo produced by human bone marrow MSC that stimulated by IFN-γ reduces the mean clinical score of EAE mice, decrease neuroinflammation, reduce demyelination, and increase the number of Treg cells^17^. Jafarinia et al. have shown that I.V. administration of EVs from human adipose tissue MSCs (hADSCs) decline the MMCS of EAE mice, inflammation score, demyelination area and proliferation of splenocytes in EAE mice^14^. Garcia et al. have demonstrated that I.V. administration of hADSCs reduce brain atrophy, inflammatory infiltrates and improve motor deficits in TMEV-induce demyelinating disease, a progressive model of MS^16^. In contrast with our study, Farrinazo et al. have shown no therapeutic effects of ASC-NVs in EAE mice. However, I.V. administration of ASC-NVs before the onset of disease significantly reduces the severity of EAE and decrease spinal cord demyelination and inflammation^15^. We suggest that the contrast between the result of Farinazzo and other studies, including our study, may be due to the dose of injected EVs which Farinazzo et al. used lower amounts of EVs than others.

MS is a T cell-mediated autoimmune disorder in CNS. Between the immune cells, Th1 and Th17 cells and their pro-inflammatory cytokines play crucial role in the pathogenesis of MS. So, a potential treatment should be able to affect these cells and their cytokines. Our RT-PCR and ELISA results showed that hUCSC-EVs treatment significantly reduce the pro-inflammatory cytokines (IFN-γ, TNF-α, and IL-17a) and increase the anti-inflammatory cytokines (IL-10 and IL-4). Since MSC-EVs have the same function as their parent cells, it is predictable that MSC-EVs can exert immunomodulatory effect. In agreement with our study, Farinazzo et al. have shown that the presence of ASC-NVs in cell culture reduce the pro-inflammatory cytokines (IFN-γ, GM-CSF, TNF-α, and IL-17a) by T cells, suggesting that ASC-NVs limit T cell activation in vitro^15^. The reduction of pro-inflammatory cytokines such as IFN-γ and IL-12p70 after treatment with hADSC-EVs has also shown in the study of Garcia et al.^16^. Riazifar et al. have also shown that in the presence of MSC-exosome, the level of several pro-inflammatory Th1 and Th17 cytokines such as IL-6, IL-12p70, IL-17AF, and IL-22 were decreased^17^. Since MSC-Exo can increase the number of Treg cells, no wonder to expect that Treg related cytokines (IL-10 and TGF-ß) also increase after MSC-Exo treatment^25,30^. The immunomodulatory effects of MSC-EVs could be mediated by targeting different immune cells^31,32^. In humoral immunity, MSC-EVs could decrease inflammation to facilitate regeneration by influencing the complement activity^33^. In cellular immunity, it has been shown that MSC-exosome could enhance infiltration of M2 over M1 macrophages by suppression of IL-1ß and TNF-α^34^. The immunomodulatory effect of MSC-EVs by increasing Treg cells has been shown in many studies. MSC-EVs have been found to increase the production of IL-10 and TGF-ß1 in PBMCs of patients with asthma and reduce inflammation in mice through increased proliferation and immunosuppressive of Tregs^35^. Increased number of Treg cells following treatment of EAE mice with MSC-EVs have been also demonstrated in the studies of Jafarinia et al. and Riazifar et al.^14,17^. MSC-EVs also mediate their immunomodulatory effect by influencing other immune cells, such as inhibition of antigen-presenting cells (APCs), suppression of Th1 and Th2 cells, and inducing the conversion of Th1 into Th2 cells^36^.

Our histological results showed a reduction in infiltrate cells in spinal cord of hUCSC-EVs-treated mice. Our results are in line with all other studies which have evaluated the effect of MSC-EVs on inflammatory infiltrate cells in MS model. Garcia et al. have shown that the number of inflammatory infiltrates was significantly lower in MSC-EVs treated mice compared to control mice in a model of progressive MS^16^. Jafarinia et al. have also shown that the inflammation score in hADSC-EV-treated groups significantly declined compared to the untreated control group^14^. We also showed that there is no significant difference between the percent of repair-associated markers, such as GFAP and MBP, in spinal cord of experimental groups. In the study of Garrcia et al., they have shown a significant reduction in MFI of GFAP and ionized calcium-binding adaptor molecule 1 (Iba1) and a significant increase in MBP 2′,3′-cyclic nucleotide-3′-phosphodiesterase (CNPase) in the brain of the TMEV-EVs mice compared to TMEV-vehicle group. However, they observed no significant difference in above mentioned markers in the spinal cord^16^. In agreement with our study, no difference have been observed in the MFI of repair-associated markers, MBP and OLIG2, in the spinal cord hADSC-EV-treated groups compared to control mice in the study of Jafarinia et al.^14^. More studies need to be done for clarifying the role of MSC-EVs on CNS repair-associated markers, such as MBP, GFAP, OLIG2, Iba1, and CNPase.

Our flow cytometry analysis for the frequency of Treg cells showed that the frequency of CD4^+^CD25^+^FoxP3^+^ Treg cells in the spleen of hUCSC-EVs-treated mice was significantly higher than EAE control mice. Our result is in line with the study of Kordelas et al. who have shown that MSC-EV treatment induced the generation of Treg cells in an allogeneic skin graft model^25^. Tamura et al. have also demonstrated that the percentage of Treg cells significantly increased following treatment with MSC-EVs on a concanavalin A-induced liver injury model^37^. Enhancing Treg function has also been shown in islet transplantation following treatment MSC-Exo^38^. In this regard, Zhang et al. have shown that MSC-Exo could induce polarization of CD4^+^ cells to CD4^+^CD25^+^Foxp3^+^ cells^39^. Based on our result and similar results regarded to increase in the frequency of Treg cells, it can be suggested that polarization or increase in the frequency of Treg cells is a mechanism by which MSC-EVs mediate their immunomodulatory effects. These cells could exert their immunomodulatory effects in a cell-to-cell manner or by producing anti-inflammatory cytokines such as IL-10 or TGF-ß^25^.

## Conclusion

Finally, we suggest that intravenous administration of hUCSC-EVs alleviate induce-EAE by reducing the pro-inflammatory cytokines, such as IL-17a, TNF-α and IFN-γ, and increasing the anti-inflammatory cytokines, IL-10 and IL-4, and also decrease the leukocyte infiltration in an animal model of MS. It seems that EVs from hUC-MSCs have the same therapeutic effects similar to EVs from other source of MSCs, such as adipose or bone marrow MSCs. Since in cell therapy, there are always concerns about malignancy, rejection, and genetic disability, hUCSC-EVs look to be an ideal approach to be studied in clinical trials of different inflammatory diseases, such as MS.

## Methods

### Isolation of hUCSC

Approximately 20 cm of umbilical cord was collected from healthy mothers after their first-term delivery. Informed patient consent was obtained. The umbilical cord was immediately transferred to the laboratory within the optimal period of 6 h. To isolate hUCSC, it was washed intensively in phosphate-buffer saline (PBS; Bioidea, Iran) and chopped into smaller 2 cm length pieces. Each of the pieces was washed gently and squeezed with curved forceps to remove blood trapped within the umbilical blood vessels. Then, sections were cut into 1–3 mm^3^ pieces and digested by collagenase type II (Sigma-Aldrich, St. Louis, MO, USA) and 0.125% trypsin in PBS for one hour. After neutralizing the enzyme by 10% fetal bovine serum (FBS; Bioidea, Iran), the cell suspension filtered through mesh. The sample was centrifuged and suspended in low glucose Dulbecco's Modified Eagle Medium (L-DMEM) (Bioidea, Iran); supplemented with 10% FBS, 100 U/ml penicillin, and 100 µg/ml streptomycin. Cells were seeded into tissue T75 culture flasks and were expanded at 37 °C and 5% CO_2_ for 48 h. The culture medium was refreshed every 3 days. Cells were passaged to three new culture flasks when cultures reached 80% confluence^10^.

### Characterization of hUCSC

To characterize hUCSC, cells were tested for surface markers by flow cytometry, being negative for CD45 (Biolegend, USA) and positive for CD73 (Biolegend, USA) and CD105 (Biolegend, USA). hUCSCs were placed in polystyrene fluorescence activated cellsorter (FACS) tubes, and labeled with peridinin chlorophyll protein/cyanin 5.5 (PerCP/Cy5.5) anti-CD45, and fluorescein isothiocyanate (FITC) anti-CD73, and phycoerythrin (PE) anti-CD105 for 30 min in dark. Data were acquired using a FACSCalibur flow cytometer and analyzed using FlowJo software (version 10, USA).

We also evaluated MSCs differentiation to adipocyte and osteocytes. For the induction of adipogenesis, hUCSCs were cultured for 2 weeks in the presence of 1 × 10^–8^ M dexamethason and 10 ng/ml insulin and stained by Oil Red O. Osteogenic induction were achieved on the culture of hUCSCs with 10 mM glycerol 2-phosphate disodium salt, 1 × 10^–8^ M dexamethasone, and 50 µg/ml l-ascorbic acid-2 phosphate for 3 weeks. Osteogenic differentiation was detected by Alizarin red staining.

### Isolation of hUCSC-EVs

hUCSC-EVs were isolated from the culture medium of hUCSCs, as previously described by Jafarinia et al.^14^. In summary, when the hUCSCs achieved to 80–90% confluence, the conventional culture medium was replaced with a medium containing 10% EV-free FBS. EV-free FBS was prepared by sequential centrifugation at 4 °C at 400 × *g* for 20 min, 20,000 × *g* for 30 min and 110,000 × *g* for 7 h followed by filtration using a 0.22 µm filter. The culture medium was collected after 48 h of culturing with medium containing 10% EV-free FBS and ultracentrifugation were used for isolating the EVs. To discard the cells, the medium was centrifuged at 4 °C at 400 × *g* for 20 min. Then, supernatants centrifuged at 20,000 × *g* for 30 min to discard cell debris. To obtain EVs, supernatant filtered by a 0.22 µm filter followed by ultracentrifugation at 110,000 × *g* for 90 min and resuspended in PBS.

### Characterization of hUCSC-EVs

The hUCSC-EVs were characterized by testing phenotype expressing markers such as CD9 and CD63 by flow cytometry, DLSand electron microscopy for size and morphology, and protein BCA assay kit (Solarbio, China) for measuring the protein concentration. For flow cytometry assay, hUCSC-EVs were resuspended in PBS and labeled with anti-CD63-coated beads (15 µg of EV/7 µl of beads, Life Technologies, Norway) overnight with gentle agitation. The beads were washed 3 times with 1% EV-depleted FBS in PBS, incubated with human IgG (Sigma-Aldrich) for 15 min at 4 °C. Then, the beads were incubated with PE-labeled with anti-CD9 and CD63 (BD Bioscience, USA) for 40 min at room temperature. Data were acquired using a FACSAria (BD Bioscience, USA) and analyzed using FlowJo software (version 10, USA). The size distribution of hUCSC-EVs was evaluated by DLS. It was performed using Zetasizer system (Malvern, UK). Before analysis, EVs were diluted in PBS at a 1:10 ratio. Data were analyzed by the Malvern software (Zetasizer Ver. 7.11). The size and morphology of hUCSC-EV were analyzed by scanning electron microscopy (KYKY-EM3200, Beijing, China)^40^. Briefly, isolated hUCSC-EV were first diluted in PBS at 1:50 ratio and placed on a sterile glass slide until dried at room temperature. Finally, the slides were observed under the scanning electron microscope.

### EAE induction and treatment protocol

Twenty, 6–8 weeks old, female C57Bl/6 mice, were purchased from Pasteur institute of Iran. Animals were kept in pathogen-free condition, climate-controlled facilities, and treated under the approval of animal’s ethical committee of Islamic Azad University, Branch Marvdasht. For inducing chronic EAE, mice were immunized subcutaneously with 200 ml emulsion containing 400 µg MOG 35–55 (100 µl; SBS Genetech Co. Ltd., Beijing, China) and complete Freund’s adjuvant containing 0.4 mg mycobacterium tuberculosis (100 µl; sigma) between the shoulder and into the flank of the mice, as previously described^14^. Mice were also injected intraperitoneally with two additional injections of pertussis toxin (250 ng) (Sigma, USA) on the day of immunization and 48 h later.

The EAE mice randomly divided into two groups (10 mice in each group) to evaluate the clinical and pathological efficacy of hUCSC-EVs. First group was EAE-induced mice that administrated I.V. with PBS on day-9 post immunization. Second group was EAE-treated mice that intravenously received 50 µg hUCSC-EVs on day-9 post immunization. The clinical score were registered according to the following scale: 0 = no symptoms; 1 = partial loss of tail tonicity; 2 = complete loss of tail tonicity; 3 = flaccid tail and abnormal gait; 4 = hind leg paralysis; 5 = hind leg paralysis with hind body paresis; 6 = hind and foreleg paralysis; and 7 = death^14^.

### Histology staining

Mice were sacrificed at day 30 post immunization. The mice spinal cord was removed after transcardial perfusion with saline at the time of sacrifice (30 DPI) and fixed in 10% formalin. After 48 h, the paraffin blocks were made and sections were cut at 5 µm on a microtome. H&E staining was used to estimate the rate of spinal cord inflammatory infiltrates. We used three section of each spinal cord region (cervical, thoracic and lumbar) for staining. For H&E staining, the paraffin slides were de-waxed, rehydrated, and immersed 1 min in hematoxylin and 3 min in eosin previous to water washes. The slides were dehydrated and mounted. The inflammation score was determined as a scale of 0–4. A score of 0 reflects no infiltrates and a score of 4 reflects the largest number of infiltrates with all the intermediate scores (1, 2 and 3)^16^.

### Immunofluorescence staining

Assessing the expression of glial fibrillary acidic protein (GFAP) and myelin basic protein (MBP) was carried out by immunofluorescence technique. After rehydration of slides in PBS, slides permeabilized with 0.1% Triton X-100 for 1 h at room temperature. Then, they were blocked for 30 min with normal bovine serum at room temperature. Sections were incubated overnight with primary goat anti-MBP monoclonal antibody (Santa Cruz Biotechnology, USA) and rabbit anti-GFAP monoclonal antibody (Abcam, UK) followed by appropriate FITC conjugate secondary antibody (Abcam, UK) for 1 h at room temperature. Finally, after 3 washes with PBS, the cells were incubated with 4′,6‐diamidino‐2‐phenylindole (DAPI) (Abcam, UK) counterstaining for 5 min and observed with fluorescence microscopy. Positive cell counting was performed by ImageJ (version 1.37; National Institute of Health)as cell number per mm^2^.

### Gene expression analysis in the CNS

Gene expression analysis was performed in the CNS of experimented groups, as previously described by Azimzadeh et al.^41^. In summary, for evaluating the inflammatory and anti-inflammatory cytokines expression, the spinal cord was extracted from experimental groups at 30 DPI. Total RNA isolation from the tissue was performed by the BioFACT™ Total RNA Prep Kit and NanoDrop spectrophotometer was used for checking RNA concentration. cDNA synthesis from 1 μg of total RNA was achieved with cDNA Reverse Transcription Kit (Bio FACT). For checking the purity of the obtained cDNA, NanoDrop was used. Real-time RT-PCR was done in duplicate using SYBR^*®*^ Green PCR Master Mix (BioFACT) for five genes such as TNF-α, IL-4, IL-10, IL-17a and IFN-γ. PCR Master Mix was contained 5 μl of SYBR Green, 1 μl of cDNA, 3.75 μl of nuclease-free water, and 0.25 μl of primer (forward and reverse mixture). The β- actin housekeeping gene was used as internal control gene for this study. Thresholds of all genes were calculated with the relative quantification method (2^−ΔΔCT^) according to standard protocol and mRNA expression fold relative to control were considered for analysis. The specific primers of the above genes were designed in our laboratory by the AlleleID software, v 7.85 (http://premierbiosoft.com). Table 2 lists the sequence of primers.

**Table 2:** The sequence of primers used in the current study.

Primer name	Sequence (5′ → 3′)
IL-4	Forward: 5′-AGTTGTCATCCTGCTCTTCTT-3′ Reverse: 5′-TGTGGTGTTCTTCGTTGCT-3′
IL-10	Forward: 5′-GCTATGCTGCCTGCTCTT-3′ Reverse: 5′-CAACCCAAGTAACCCTTAAAGT-3′
IL-17a	Forward: 5′-GACTCTCCACCGCAATGA-3′ Reverse: 5′-ACACCCACCAGCATCTTC-3′
IFN-γ	Forward: 5′-AAAGAGATAATCTGGCTCTGC-3′ Reverse: 5′-GCTCTGAGACAATGAACGCT-3′
TNF-α	Forward: 5′-GTGGAACTGGCAGAAGAG-3′ Reverse: 5′-TTGAGAAGATGATCTGAGTGT-3′
β-actin	Forward: 5′-GGCTGTATTCCCCTCCATCG-3′ Reverse: 5′-CCAGTTGGTAACAATGCCATGT-3′

*IFN* interferon, *IL* interleukin, *TNF* tumor necrosis factor.

### Splenocytes preparation

For splenocyte preparation, we used a protocol which have been described by Jafarinia et al.^14^. In summary, after spleen isolation, a cell suspension was prepared in Roswell Park Memorial Institute (RPMI-1640) medium (Bioidea, Iran) by passing the small pieces of tissue through a cell strainer (70 µm) using the plunger end of a syringe. The cell suspension was centrifuged at 500 × *g* for 5 min at 4 °C and the red blood cells (RBC) were lysed by the addition of a lysis buffer (Cytomatingen, Iran) for 2 min at 37 °C. After adding at least 30 ml of RPMI, the cell suspension was centrifuged at 500 × *g* for 5 min at 4 °C. Supernatants discarded and the cell pellet suspended in complete RPMI containing 10% FBS (Bioidea, Iran) and 1% penicillin/streptomycin (Bioidea, Iran).

### Cytokine assay

Obtained splenocytes were cultured (2 × 10^6^ cells/well) in 24-well culture plate and stimulated with 10 μg/ml MOG for 48 h. Then, the supernatants were collected and the amount of different cytokines (IL-10, IL-4, IL-17a, IFN-γ, and TNF-α) was measured by enzyme-linked immunosorbent assay (ELISA) kits (eBioscinece, San Diego, CA, USA) according to the manufacture’s protocol. All samples were performed in duplicate.

### Flow cytometry analysis for Treg

For evaluating the frequency of Treg cells, splenocytes were first surface stained with PE anti-mouse CD4 (Biolegend) and FITC anti-mouse CD25 (Biolegend). After permeabilization with Foxp3 buffer set (BD Biosciences), cells were incubated with PerCP/Cy5.5 anti-mouse Foxp3 for intracellular staining. Finally, Data were acquired and analyzed with FACSCalibur flow and flowJo software (Version 10).

### Animals’ guideline

Animals were kept in pathogen-free condition, climate-controlled facilities, and treated under the approval of animal’s ethical committee of Islamic Azad University, Branch Marvdasht. For induction of EAE, the mice were anesthetized with proper dose of xylazine (10 mg/kg) and ketamine (100 mg/kg) and then were injected by MOG, CFA, and pertussis toxin. For sacrificing the mice, they were anesthetized with xylazine (10 mg/kg) and ketamine (100 mg/kg) and then tissues were removed^14^.

### Ethics declarations

#### Approval for animal experiments

The study was approved by the ethical committee of Marvdasht branch, Islamic Azad University and all experiments were performed in accordance with ARRIVE guideline and regulations. Our protocols adhered to the guidelines/regulations/directives for experimental on animals.

#### Approval for human experiments

The study was approved by the ethical committee of Marvdasht branch, Islamic Azad University and informed consent was obtained from all participants and/or their legal guardians. All experiments were performed in accordance with declaration of Helsinki guideline and regulations.

### Statistical analysis

Statistical analysis was analyzed by IBM SPSS, Version 24.0 (IBM Crop., Armonk, NY, USA). The normal distribution of data was checked by the Kolmogorov–Smirnov Z test. Independent t-test was used to compare the groups with normal distribution and the Mann–Whitney tests in the case of non-normal distribution. All data in the study are shown as mean ± standard deviation (SD). GraphPad Prism 8 software (GraphPad Software, Inc., USA) was used to prepare the graphs. P-values less than 0.05 were considered statistically significant.

## Data Availability

The dataset of the current study are available from the corresponding author on reasonable request.
